# Epidemiological characteristics and the entire evolution of coronavirus disease 2019 in Wuhan, China

**DOI:** 10.1186/s12931-020-01525-7

**Published:** 2020-10-08

**Authors:** Dongming Wang, Jing Cai, Tingming Shi, Yang Xiao, Xiaobing Feng, Meng Yang, Wenzhen Li, Wei Liu, Linling Yu, Zi Ye, Tao Xu, Jixuan Ma, Mingyan Li, Weihong Chen

**Affiliations:** 1 Department of Occupational & Environmental Health, School of Public Health, Tongji Medical College, Huazhong University of Science and Technology, Wuhan, 430030 Hubei China; 2 Key Laboratory of Environment and Health, Ministry of Education & Ministry of Environmental Protection, and State Key Laboratory of Environmental Health (Incubating), School of Public Health, Tongji Medical College, Huazhong University of Science and Technology, Wuhan, 430030 Hubei China; 3 Institute of Preventive Medicine Information, Hubei Provincial Center for Disease Control and Prevention, Wuhan, 430079 Hubei China; 4 Division of Human Resources, Science and Education, Hubei Provincial Center for Disease Control and Prevention, Wuhan, 430079 Hubei China; 5 Department of Social Medicine and Health Management, School of Public Health, Tongji Medical College, Huazhong University of Science and Technology, Wuhan, 430030 Hubei China

**Keywords:** Coronavirus disease 2019 (COVID-19), Epidemiological characteristics, Evolution, Non-pharmaceutical interventions

## Abstract

**Background:**

Coronavirus Disease 2019 (COVID-19) spread rapidly around the world. We aimed to describe the epidemiological characteristics and the entire evolution of COVID-19 in Wuhan, and to evaluate the effect of non-pharmaceutical intervention by the government.

**Methods:**

The information of COVID-19 cases until Mar 18, 2020 in Wuhan were collected from the national infectious disease surveillance system in Hubei province.

**Results:**

A total of 49,973 confirmed cases were reported until Mar 18, 2020 in Wuhan. Among whom, 2496 cases died and the overall mortality was 5.0%. Most confirmed cases (25,619, 51.3%) occurred during Jan 23 to Feb 4, with a spike on Feb 1 (new cases, 3374). The number of daily new cases started to decrease steadily on Feb 19 (new cases, 301) and decreased greatly on Mar 1 (new cases, 57). However, the mortality and the proportion of severe and critical cases has been decreasing over time, with the lowest of 2.0 and 10.1% during Feb 16 to Mar 18, 2020, respectively. The percentage of severe and critical cases among all cases was 19.6%, and the percentage of critical and dead cases aged over 60 was 70.1 and 82.0%, respectively.

**Conclusion:**

The number of new cases has dropped significantly after the government taking the isolation of four types of personnel and the community containment for 14 days. Our results indicate that the mortality and proportion of severe and critical cases gradually decreased over time, and critical and dead cases are more incline to be older individuals.

## Background

Coronavirus Disease 2019 (COVID-19) has been named by the World Health Organization (WHO) since Feb 11, 2020 [1], and the WHO announces a pandemic of COVID-19 on Mar 11, 2020 [2]. Common clinical manifestations of COVID-19 include cough, fever, and pneumonia, and the pathogen of this disease is severe acute respiratory syndrome coronavirus 2 (SARS-CoV-2) [3], which is similar to SARS-CoV. There are more than 570, 000 confirmed cases and more than 26,000 deaths have been reported in 199 countries and regions on Mar 28, 2020 by WHO [4]. Rapidly spreading and growing cases of COVID-19 have become major risks for global health.

The pneumonia cases of unknown causes were appeared in December 2019 in Wuhan, China and pathogenic pathogens were identified as 2019-nCoV [5, 6], a new coronavirus on Jan 7, 2020. Subsequently, detection methods were developed based on the genetic sequence of the virus. By mid-January in 2020, some doctors noticed this new pneumonia could spread from person to person, which quickly caused great concern [7, 8]. In order to avoid the spread of COVID-19 and block transmission to other regions, the government not only asked all citizens to stay at home and interrupted public traffic in the city, but also suspended all transport links and blocked the roads out of the Wuhan city on 23 January, 2020 [9], covering approximately 10 million populations in Wuhan [10, 11]. In response to the outbreak, other non-pharmaceutical interventions including shelter hospitals and community containment measures were also enforced during the evolution of COVID-19 in Wuhan [12]. These intervention measures should play important role in stopping the spread of the disease. As for Mar 18, 2020, the new suspected and confirmed cases of COVID-19 were both reported to be zero in Wuhan [13]. Although some models have analyzed the prevalence and spread of COVID-19 in Wuhan [14, 15], the actual incidence information is still not very clear. In particular, to what extent did the non-pharmaceutical interventions in Wuhan play the roles and when did they start to work? All these are highly concerned and expected by COVID-19 epidemic areas around the world.

In this study, we described the full spectrum of epidemiological characteristics of confirmed cases with COVID-19 in Wuhan from the beginning to Mar 18, 2020 according to the data from the national infectious disease surveillance system. Meanwhile, we also evaluated the effect of the intervention measures implemented by the government.

## Methods

### Source of data

COVID-19 is classified as a Class B infectious disease on Jan 20, 2020 in China and all infectious cases should be reported immediately through the infectious disease information system according to legal requirements. All data of COVID-19 cases until Mar 18, 2020 in Wuhan in this study were extracted from the national infectious disease surveillance system, including age, sex, the date of illness onset, the date of diagnosis, clinical severity, etc. The first-diagnose physician reports case information in a fixed infectious disease report card format. The completeness and accuracy is verified by public health physician in the hospital and then reported to the infectious disease surveillance system. The county-level, city-level and provincial-level CDC completed three reviews and confirmations of the report card within 2 h, to ensure the accuracy of the data. Before analysis, we removed personally identifiable information to protect personal privacy.

### Confirmed case definition

Diagnosis of confirmed COVID-19 in our study was conducted according to the diagnostic and treatment scheme for COVID-19 released by the National Health Commission of China, including laboratory confirmed cases and clinical diagnosed cases [16]. A laboratory confirmed case was defined if a patient had clinical feature (fever, respiratory symptom, etc), a clear epidemiological history and a positive test of SARS-CoV-2 virus or high-throughput sequencing of nasal and pharyngeal swab specimens. In brief, throat swab samples from patients were collected for extracting COVID-19 RNAs testing. The total RNA was extracted within 2 h using the respiratory sample RNA isolation kit (Zhongzhi, Wuhan, China), and tested with real-time reverse transcription polymerase chain reaction (RT-PCR). A cycle threshold value (Ct-value) less than 37 was defined as a positive result, and a Ct-value of 40 or more was defined as a negative one [17]. A clinical diagnosed case was defined as a patient with corresponding clinical symptoms, epidemiological history and the imaging characteristics of pneumonia. Meanwhile, the severity status was categorized as mild, moderate, severe, and critical when they were diagnosed.

### Classification of four time periods

**Fig. 1:**
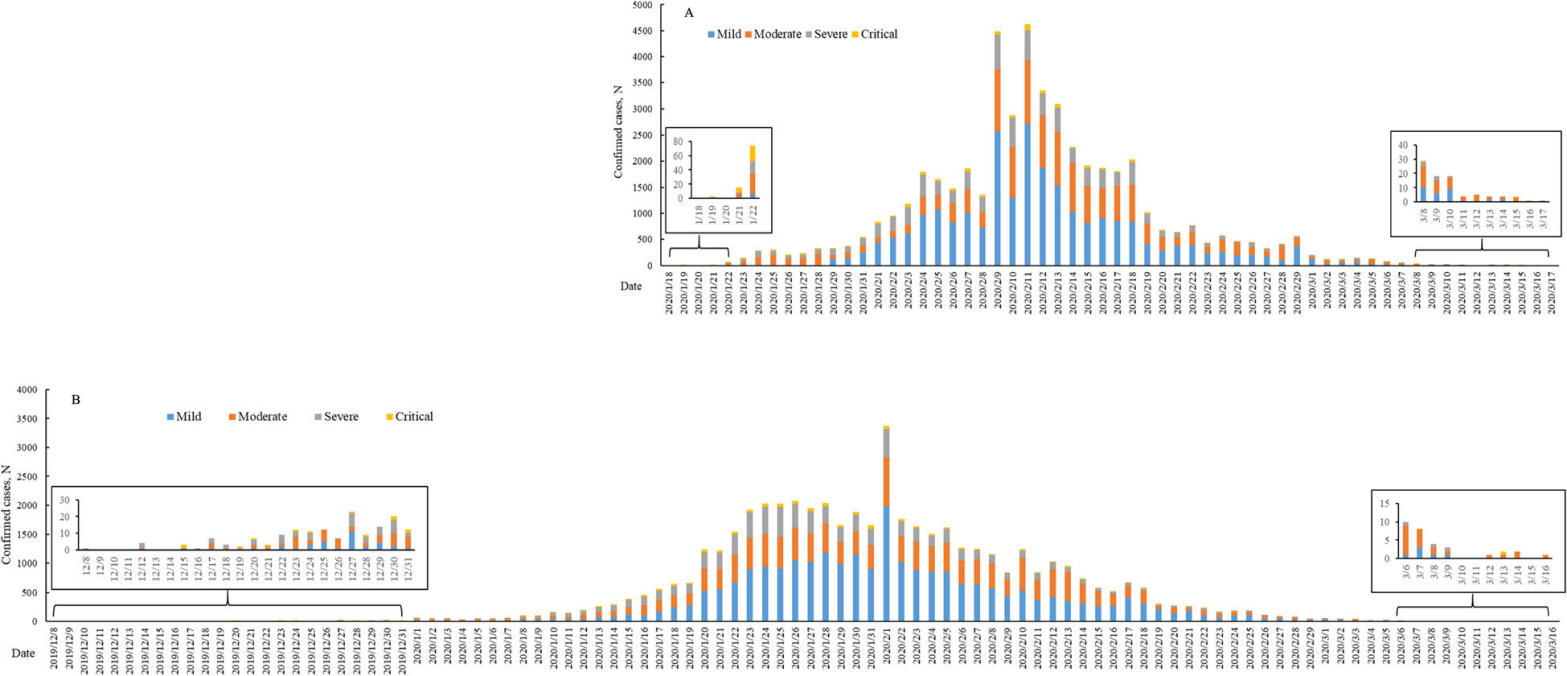
The number of daily new confirmed cases in Wuhan until Mar 18, 2020: **a**, by the date of diagnosis; **b**, by the date of illness onset

To better reflect the epidemiological characteristics of the COVID-19 and corresponding interventions, we classified the outbreak into four periods based on dates of the key implementation of control measures in Wuhan (**e-**Fig. 1). The first period is from the first case to Jan 22, 2020, the day before Wuhan isolation. The second period is from Jan 23 to Feb 4, 2020. At Feb 5, centralized treatment and isolation of “four types of personnel” were completed in the city, and patients were admitted to shelter hospitals and Huoshenshan hospital. The four types of personnel are: confirmed cases, suspected cases, cases with fever that cannot be ruled out, and close contacts of confirmed cases. The third period is from Feb 5 to Feb 15, 2020, as Feb 16 is the date that all the communities were closed in Wuhan and all the people have to stay at home and not go out of the community without special demands. The final period is from Feb 16 to Mar 18, 2020, the date that the new suspected and confirmed cases of COVID-19 in Wuhan were both reported to be zero.

### Statistical analysis

We described the epidemiological characteristics of the COVID-19 and corresponding interventions according to the four periods. We also described the basic information of these confirmed cases in different districts of Wuhan, and used the map of Wuhan to show the change of the new and total number of confirmed cases. All the data were reported according to the date of illness onset. The date of illness onset was defined as the time of self-reported first symptom of infected cases. The diagnosis duration was defined as duration from the date of illness onset to the date of diagnosis, and the death duration was defined as duration from the date of illness onset to the date of death for dead cases. Finally, spearman correlation test was used to explore the association of different severity types of cases with the population in different districts of Wuhan. All statistical analyses were performed using SAS 9.4 software (SAS Institute, Cary, NC) or R version 3.5.1 (R Core Team 2016).

## Results

A total of 49,973 confirmed cases were included in our analysis. The number of confirmed cases in Wuhan during four periods is showed in Table 1. Among them, 47.3% were male, and 77.2% aged between 41 and 80 years old. As of Mar 18, 2020, a total of 2496 cases died and the overall mortality was 5.0%. The median time from illness onset to diagnosis and death was 10.7 and 16.0 days, respectively.

**Table 1:** Characteristics of confirmed cases in Wuhan until Mar 18, 2020

Characteristics	Before 1.23	1.23–2.4	2.5–2.15	2.16–3.18	Total
Total, *n*	8841	25,619	11,583	3930	49,973
Sex, *n* (%)					
Male	4170 (47.2)	12,279 (47.9)	5463 (47.2)	1748 (44.5)	23,660 (47.3)
Female	4671 (52.8)	13,340 (52.1)	6120 (52.8)	2182 (55.5)	26,313 (52.7)
Age, mean ± SD	54.8 ± 15.0	54.4 ± 15.6	52.8 ± 17.7	50.9 ± 21.0	53.8 ± 16.5
Age group, *n* (%)					
0- ≤ 18	29 (0.3)	198 (0.8)	322 (2.8)	263 (6.7)	812 (1.6)
19- ≤ 40	1761 (19.9)	5216 (20.3)	2641 (22.8)	977 (24.9)	10,595 (21.2)
41- ≤ 60	3524 (39.9)	10,243 (40.0)	4340 (37.4)	1357 (34.5)	19,464 (38.9)
61- ≤ 80	3203 (36.2)	9000 (35.1)	3701 (32.0)	961 (24.4)	16,865 (33.8)
≥ 81	324 (3.7)	962 (3.8)	579 (5.0)	372 (9.5)	2237 (4.5)
Diagnosis duration, median (Q1-Q3)*	20.8 (14.5–26.38)	11.8 (8.5–16.4)	5.63 (3.4–8.6)	2.8 (1.4–6.4)	10.7 (5.8–16.6)
Number of death, *n*	744	1316	356	80	2496
Mortality, %	8.4	5.1	3.1	2.0	5.0
Death duration, median (Q1-Q3)^&^	21 (14–28)	14 (9–22)	11 (6–18)	10 (4.5–15.0)	16 (10–23)
Clinical severity^#^, *n* (%)					
Mild	3133 (35.4)	13,789 (53.8)	5418 (46.8)	2162 (55.0)	24,502 (49.0)
Moderate	2780 (31.4)	6623 (25.9)	4533 (39.1)	1365 (34.7)	15,301 (30.6)
Severe	2227 (25.2)	4476 (17.5)	1417 (12.2)	359 (9.1)	8479 (17.0)
Critical	444 (5.0)	640 (2.5)	170 (1.5)	38 (1.0)	1292 (2.6)

*Diagnosis duration means duration from the date of illness onset to the date of diagnosis

^&^Death duration means duration from the date of illness onset to the date of death

^#^ A total of 399 cases were reported with miss information

**Fig. 2:**
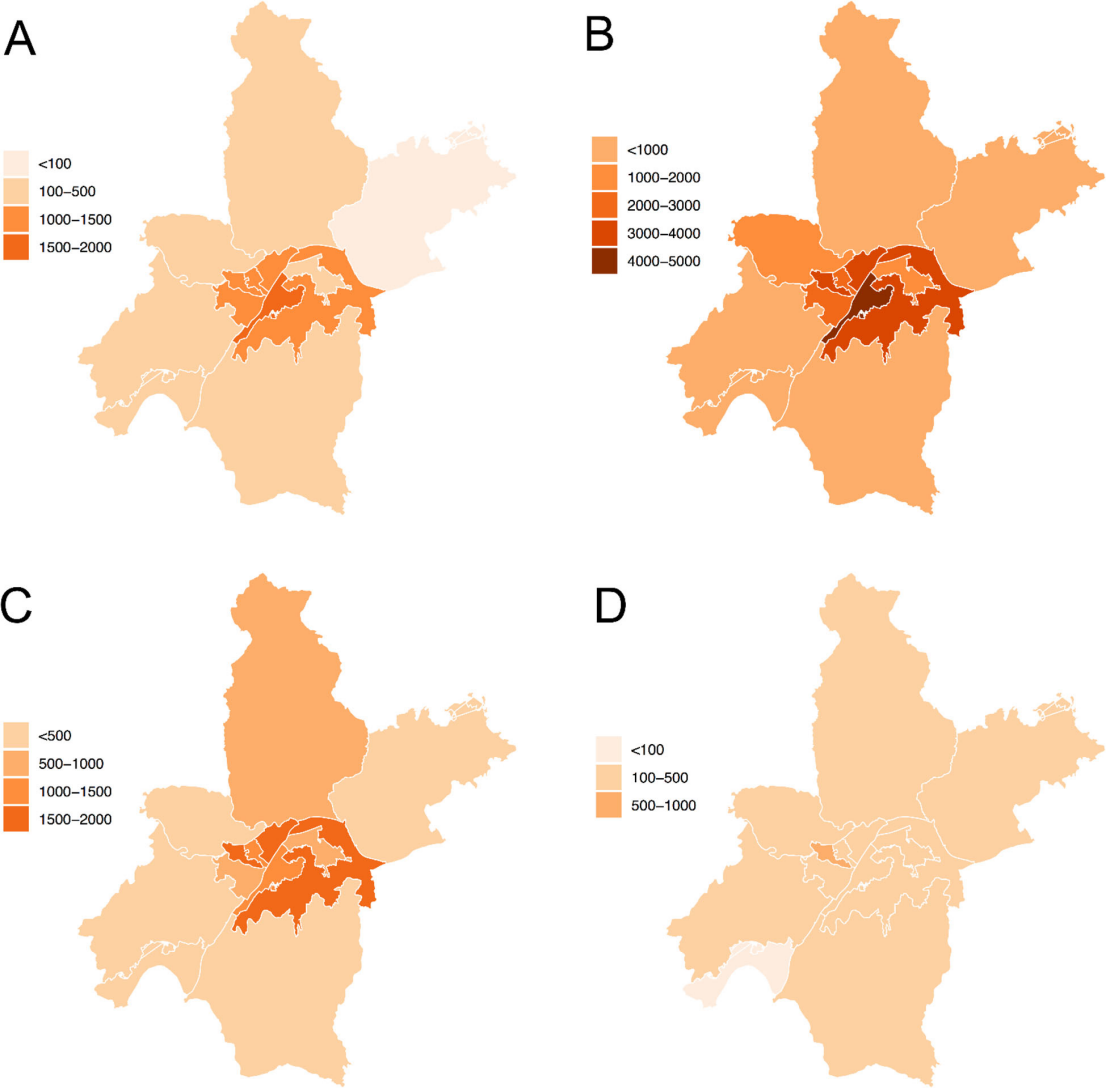
The number of new confirmed cases in Wuhan: **a**, before Jan 23, 2020; **b**, from Jan 23 to Feb 4, 2020; **c**, from Feb 5 to Feb 15, 2020; **d**, from Feb 16 to Mar 18, 2020

The number of daily new and total confirmed cases are showed in Fig. 1 and **e-**Fig. 2, respectively. In Fig. 1, the number of daily new confirmed cases was described by the date of illness onset and the date of diagnosis simultaneously. According to the date of illness onset, the number of new confirmed cases was 8841, 25,619, 11,583, and 3930 in the first to fourth period. And most cases (25,619/49,973, 57.0%) occurred in the second period (during Jan 23 to Feb 4), with a spike on Feb 1 (new cases, 3374). In addition, the mortality of COVID-19 has been decreasing since Jan 23, 2020, which was 8.4, 5.1, 3.1 and 2.0% in the first to fourth period, respectively.

The characteristic of confirmed cases in different districts in Wuhan is revealed in Table 2. The top five districts of confirmed cases were Wuchang (7484, 15.0%), Hongshan (6990, 14.0%), Qiaokou (6863, 13.7%), Jiangan (6570, 13.2%) and Jianghan (5199, 10.4%), and the last one was Xinzhou (1073, 2.2%). The differences of the sexual and age proportion, diagnosis duration were statistically significant among different districts (all *P* < 0.001). In addition, the change of the number of new confirmed cases and total confirmed cases during four periods in different districts are showed in Fig. 2 and **e-Fig.** **3**, respectively. The highest new cases occurred in Wuchang (new cases, 4240) and Hongshan (new cases, 3853) during the second period (Jan 23 to Feb 4).

**Table 2:** Characteristics of confirmed cases in different districts in Wuhan until Mar 18, 2020

District	N	Male, *n* (%)	Age, mean ± SD	Age group, n (%)					Diagnosis duration,
				0- ≤ 18	19- ≤ 40	41- ≤ 60	61- ≤ 80	≥81	median (Q1-Q3)
Jiangan	6570	3201 (48.7)	54.9 ± 16.7	101 (1.5)	1289 (19.6)	2467 (37.6)	2351 (35.8)	362 (5.5)	9.5 (4.8–15.5)
Jianghan	5199	2435 (46.8)	55.7 ± 16.0	47 (0.9)	952 (18.3)	1983 (38.1)	1912 (36.8)	305 (5.9)	10.7 (5.9–17.5)
Qiaokou	6863	2850 (41.5)	53.9 ± 15.5	71 (1.0)	1399 (20.4)	2911 (42.4)	2208 (32.2)	274 (4.0)	11.7 (6.9–16.8)
Hanyang	4691	2200 (46.9)	53.9 ± 16.1	69 (1.5)	1009 (21.5)	1789 (38.1)	1648 (35.1)	176 (3.8)	12.7 (8.4–18.5)
Wuchang	7484	3582 (47.9)	55.7 ± 16.5	93 (1.2)	1412 (18.9)	2734 (36.5)	2823 (37.7)	422 (5.7)	10.7 (6.0–16.4)
Qingshan	2788	1289 (46.2)	57.7 ± 15.9	29 (1.0)	406 (14.6)	985 (35.3)	1197 (42.9)	171 (6.2)	10.6 (5.7–15.8)
Hongshan	6990	3446 (49.3)	52.3 ± 16.6	121 (1.7)	1720 (24.6)	2717 (38.9)	2162 (30.9)	270 (3.9)	10.6 (5.7–16.4)
Dongxihu	2465	1158 (46.9)	52.5 ± 16.1	58 (2.3)	531 (21.5)	1042 (42.3)	765 (31.1)	69 (2.8)	11.0 (6.7–16.0)
Hannan	1094	556 (50.8)	51.4 ± 16.9	30 (2.7)	279 (25.5)	418 (38.2)	332 (30.4)	35 (3.2)	10.5 (4.8–16.6)
Caidian	1417	712 (50.2)	51.8 ± 16.5	22 (1.5)	360 (25.4)	563 (39.7)	423 (29.9)	49 (3.5)	7.7 (2.8–14.4)
Jiangxia	1213	603 (49.7)	47.9 ± 18.6	64 (5.2)	365 (30.1)	450 (37.1)	294 (24.3)	40 (3.3)	10.6 (5.4–16.6)
Huangpi	2126	1074 (50.5)	49.2 ± 16.9	74 (3.5)	566 (26.6)	914 (43.0)	518 (24.4)	54 (2.5)	10.6 (4.7–16.8)
Xinzhou	1073	554 (51.6)	47.8 ± 15.9	33 (3.1)	307 (28.6)	491 (45.8)	232 (21.6)	10 (0.9)	9.6 (4.7–12.9)
*P* value		< 0.0001^a^	< 0.0001^b^	< 0.0001^a^					< 0.0001^c^

*Diagnosis duration means duration from the date of illness onset to the date of diagnosis

^a^ Chi-square test, ^b^ ANOVA, ^c^ Non- parametric test

The characteristic of different severity types of cases is revealed in **e-**Table 1. Most of cases (39,803, 79.6%) were mild or moderate, and 19.5% of cases were severe and critical. Critical and dead cases were more incline to be male, and aged between 61 and 80 years old. The percentage of critical and dead cases aged over 60 was 70.1 and 82.0%, respectively. In addition, the duration from the date of illness onset to the date of diagnosis of severe (median, 12.6 days) and critical (median, 12.7 days) cases was also longer than other cases (median, 10.5 days for mild and 9.8 days for moderate). Distribution of different severity types of cases in each district is also showed in Table 3. The top five districts of severe and critical cases were Jiangan (1901, 29.1%), Qingshan (687, 24.8%), Wuchang (1671, 22.6%), Xinzhou (238, 22.2%), and Jianghan (950, 18.6%), and the last one was Jiangxia (104, 9.0%). The number of daily new and total confirmed cases with different types of cases by sex and age group are revealed in **e-Fig.** **4****,** **5****,** **6****,** **7**. The distribution of confirmed cases was almost similar among these subgroups, and the percentage of critical cases among aged equal to and over 81 years old (184/2237, 8.2%) was more than other age groups (0.2, 0.6, 1.6 and 4.3% for 0- ≤ 18, 19- ≤ 40, 41- ≤ 60 and 61- ≤ 80 years old). In addition, the peak of growth curve among aged below and equal to 18 was later than others, with a spike on Feb 18, 2020 (new cases, 56). Finally, association of different severity types of cases with the population in different districts were also conducted (**e-**Table 2), and we found that confirmed cases (including death) were correlated with population density.

**Table 3:** Number of different severity types of infected individuals in Wuhan until Mar 18, 2020

District	Confirmed, *n*	Mild, *n* (%)	Moderate, *n* (%)	Severe, *n* (%)	Critical, *n* (%)	Death, *n*	Mortality, %
Total	49,973	24,502	15,301	8479	1292	2496	5.0
Jiangan	6570	2159 (33.1)	2466 (37.8)	1581 (24.2)	320 (4.9)	336	5.1
Jianghan	5199	2567 (50.2)	1592 (31.2)	846 (16.6)	104 (2.0)	307	5.9
Qiaokou	6863	3621 (53.0)	2047 (29.9)	1027 (15.0)	142 (2.1)	274	4.0
Hanyang	4691	3132 (67.0)	760 (16.3)	704 (15.1)	78 (1.7)	262	5.6
Wuchang	7484	2734 (36.9)	3011 (40.6)	1488 (20.1)	183 (2.5)	446	6.0
Qingshan	2788	1384 (50.0)	700 (25.3)	601 (21.7)	86 (3.1)	190	6.8
Hongshan	6990	3484 (50.2)	2323 (33.5)	968 (14.0)	165 (2.4)	246	3.5
Dongxihu	2465	1589 (64.6)	434 (17.7)	385 (15.7)	51 (2.1)	141	5.7
Hannan	1094	698 (64.0)	197 (18.1)	166 (15.2)	30 (2.8)	58	5.3
Caidian	1417	1037 (73.2)	142 (10.0)	194 (13.7)	43 (3.0)	57	4.0
Jiangxia	1213	327 (28.3)	725 (62.7)	81 (7.0)	23 (2.0)	42	3.5
Huangpi	2126	1293 (61.3)	549 (26.0)	212 (10.1)	55 (2.6)	90	4.2
Xinzhou	1073	477 (44.6)	355 (33.2)	226 (21.1)	12 (1.1)	47	4.4

## Discussion

In our study, we described the epidemiological characteristics of 44,973 confirmed cases in Wuhan till Mar 18, 2020. The period covering the entire evolution of COVID-19, as the reported number of new suspected and confirmed cases both decreased to zero on Mar 18, 2020. The results of this study are helpful in understanding the spread of COVID in a relatively closed population and can evaluate the effects of non-pharmaceutical interventions conducted in Wuhan city.

We classified the entire outbreak into four periods according to three important time points that blocked the spread of COVID-19. In the first period, a total of 8841 cases were reported and Wuhan adopted isolation on Jan 23, 2020. Our previous research founded that this intervention greatly reduced the spread of disease to other provinces in China [18]. In the second period (Jan 23 to Feb 4), we observed rapidly increasing number of daily new cases in Wuhan, from 8841 cases during the first period to 25,619 cases during the second period. These data indicated an outbreak of COVID-19 in Wuhan. The main reason is that Wuhan blocked the traffic out of the city, but the cases in Wuhan city were not well isolated. At that time, Wuhan adopted self-isolation of all cases and close contacts at home because so many cases appeared in short time. But nobody can guarantee the effectiveness of these isolations, or guarantee those cases actually stay at home. Rising number of cases indicated poor isolation and the government have to adopt centralized treatment and isolation of “four types of personnel” on Feb 5. The four types of personnel are: confirmed cases, suspected cases, cases with fever that cannot be ruled out, and close contacts of confirmed cases. New hospital such as Huosheshan hospital and more than 10 shelter hospitals were established to place cases and many hotels were also used to finish isolation. In the third period (Feb 5 to Feb 15), we observed that the number of daily new cases started to decrease steadily on Feb 19 (301 cases). Most studies suggested that latency of COVID-19 were 7 to 14 days [19, 20] and it was exactly 14 days from Feb 5 (complete the isolation of four types personnel) to Feb 19. In addition, we observed that the number of cases fell on Mar 1 (57 cases), which is 14 days after Feb 16, 2020 (524 cases), the date that all the communities and shops were closed in Wuhan. The trend of characteristics and effect of these intervention was similar when the number of cases were calculated according to the date of diagnosis, the spike of the number of confirmed cases according to the date of diagnosis was just later than that of the date of illness onset. The delayed phenomenon may be related with the shortage of detection kit and detectability of professional institutes in the early stage. All these intervention measures conducted by the government are confirmed to be greatly effective in stopping the spread of COVID-19 in our study.

At present, COVID-19 has attacked populations all over the world, the current number of COVID-19 infected cases have far more surpassed the previous two diseases: severe acute respiratory syndrome (SARS) and Middle East respiratory syndrome (MERS) [21, 22], and the situation may be even worse than those two diseases, because the growth trend of infected cases does not seem to be controlled effectively [6]. The intervention measures against COVID-19 taken by Wuhan may have implications for other countries. For instance, isolation of cities has been adopted by Italy, Spain and etc.; shelter hospitals were also conducted in Italy, South Korea and etc.

We also analyzed the characteristics of confirmed cases with different severity status. And we found that critical and dead cases were more incline to be older individuals, though more confirmed cases were middle-aged patients. The results were similar with Arentz’s study [23]. They found that the mean age of 21 critical cases in Washington State was 70 years old (range, 43–92 years). However, our study provided more information with the characteristics of all types of cases during the entire evolution of COVID in Wuhan. The older age of critical and dead cases may be related with the hypoimmunity and more other basic diseases of these population [17, 19]. The findings remind us to pay more attention to older infected cases. In addition, we also found that different severity types of confirmed cases were associated with population density. The results confirmed that population density played an important role in the spread of COVID-19.

We noticed that the mortality rate gradually decreased over time. It is interesting that the proportion of severe and critical cases in third (13.7%) and fourth (10.1%) periods are also significantly lower than those in the first (30.2%) and second (20.0%) periods. Several reasons are contributed to this phenomenon. Firstly, the diagnosis duration is greatly shortened, which could be verified in our study (from 20.8 days in the first period to 2.8 days to the fourth period). Secondly, the cognition of the virus and the experience of the physicians have been improved; meanwhile, the use of Huoshenshan hospital also plays a role, as Huoshenshan hospital mainly focus on the treatment of severe and critical cases. Finally, the transmission ability of the virus has been weakened with the process of the COVID-19.

The limitations should also be acknowledged. First, we got our conclusion based on the data collected from the national infectious disease surveillance system. Second, some recall bias was inevitable, as the data and the four periods in our study were reported according to the date of illness onset. However, the trend of characteristics and effects of these interventions were similar when the number of cases were calculated according to the date of diagnosis.

## Conclusion

In conclusion, we observe the entire outbreak process of COVID-19 in Wuhan city. It takes 25 days from identified pathogenic pathogens to the peak. The number of new cases has dropped significantly after the government taking the isolation of four types of personnel and the community containment for 14 days. Our results indicate that the mortality and proportion of severe and critical cases gradually decreased over time, and critical and dead cases are more incline to be older individuals.

## Supplementary information

**Additional file 1: e-Table 1.** Characteristics of different severity types of cases in Wuhan until Mar 18, 2020. **e-Table 2.** Association of different severity types of cases with the population in Wuhan. **e-Figure 1.** Dates of discovery of COVID-19, and of the key implementation of control measures in Wuhan. **e-Figure 2.** The number of daily total confirmed cases in Wuhan until Mar 18, 2020. **e-Figure 3.** The number of total confirmed cases in Wuhan: A, before Jan 23, 2020; B, until Feb 4, 2020; C, until Feb 15, 2020; D, until Mar 18, 2020. **e-Figure 4.** The number of daily new confirmed cases by sex in Wuhan until Mar 18, 2020: (A) male; (B) female. **e-Figure 5.** The number of daily new confirmed cases by age group in Wuhan until Mar 18, 2020: (A) 0- ≤ 18; (B) 19- ≤ 40; (C) 41–60; (D) 61–80; (E) ≥81. **e-Figure 6.** The number of daily total confirmed cases by sex in Wuhan until Mar 18, 2020: (A) male; (B) female. **e-Figure 7.** The number of daily total confirmed cases by age group in Wuhan until Mar 18, 2020: (A) 0- ≤ 18; (B) 19- ≤ 40; (C) 41–60; (D) 61–80; (E) ≥81.

## Data Availability

The datasets used and/or analysed during the current study are available from the corresponding author on reasonable request.

## Abbreviations

COVID-19: Coronavirus Disease 2019
WHO: World Health Organization
SARS-CoV-2: Severe acute respiratory syndrome coronavirus 2
RT-PCR: Real-time reverse transcription polymerase chain reaction
SARS: Severe acute respiratory syndrome
MERS: Middle East respiratory syndrome
