# Supplementary material for: Epidemiological characteristics and the entire evolution of coronavirus disease 2019 in Wuhan, China
Source: Respir Res. 2020 Oct 8;21:257. doi: 10.1186/s12931-020-01525-7 (PMC7542568; doi:10.1186/s12931-020-01525-7)

**e-Table 1 Characteristics of different severity types of cases in Wuhan until Mar 18, 2020**

| Characteristics | Mild | Moderate | Severe | Critical | Death |
| --- | --- | --- | --- | --- | --- |
|  |  |  |  |  |  |
| Total, n | 24,502 | 15,301 | 8,479 | 1,292 | 2,496 |
| Sex, n (%) |  |  |  |  |  |
| Male | 11,448 (46.7) | 7,048 (46.1) | 4,234 (49.9) | 741 (57.4) | 1,607 (64.4) |
| Female | 13,054 (53.3) | 8,253 (53.9) | 4,245 (50.1) | 551 (42.6) | 889 (35.6) |
| Age, mean ± SD | 51.6 ± 16.2 | 52.8 ± 16.4 | 60.4 ± 15.4 | 65.8 ± 13.9 | 70.2 ± 12.0 |
| Age group, n (%) |  |  |  |  |  |
| 0-≤18 | 515 (2.1) | 259 (1.7) | 29 (0.3) | 2 (0.2) | 1 (0.0) |
| 19-≤40 | 5,965 (24.3) | 3,430 (22.4) | 1,046 (12.3) | 62 (4.8) | 45 (1.8) |
| 41-≤60 | 10,080 (41.2) | 6,193 (40.5) | 2,694 (31.8) | 322 (24.9) | 404 (16.2) |
| 61-≤80 | 7,233 (29.5) | 4,830 (31.6) | 3,970 (46.8) | 722 (55.9) | 1,534 (61.5) |
| ≥81 | 709 (2.9) | 589 (3.8) | 740 (8.8) | 184 (14.2) | 512 (20.5) |
| Diagnosis duration, median (Q1-Q3)* | 10.5  (5.6-15.6) | 9.8  (5.5-16.6) | 12.6  (7.8-17.8) | 12.7 (7.7-18.8) | 11.4 (6.7-16.7) |

*Diagnosis duration means duration from the date of illness onset to the date of diagnosis.

**e-Table 2 Association of different severity types of cases with the population in Wuhan**

|  | Confirmed | Mild | Moderate | Severe | Critical | Death |
| --- | --- | --- | --- | --- | --- | --- |
| **Population^#^** |  |  |  |  |  |  |
| Population density, per square kilometers | 0.70* | 0.68* | 0.62* | 0.75* | 0.69* | 0.77* |
| Number of households, per household | 0.48 | 0.40 | 0.62* | 0.49 | 0.48 | 0.38 |
| Number of residents in the end of 2017, per person | 0.47 | 0.39 | 0.60* | 0.48 | 0.46 | 0.37 |
| Number of permanent residents in 2017, per 10,000 persons | 0.54* | 0.37 | 0.65* | 0.49 | 0.52 | 0.37 |

#Data coming from yearbook of Wuhan population statistics in 2018; Using Spearman Correlation Test; **P*<0.05

**e-Figure 1** Dates of discovery of COVID-19, and of the key implementation of control measures in Wuhan


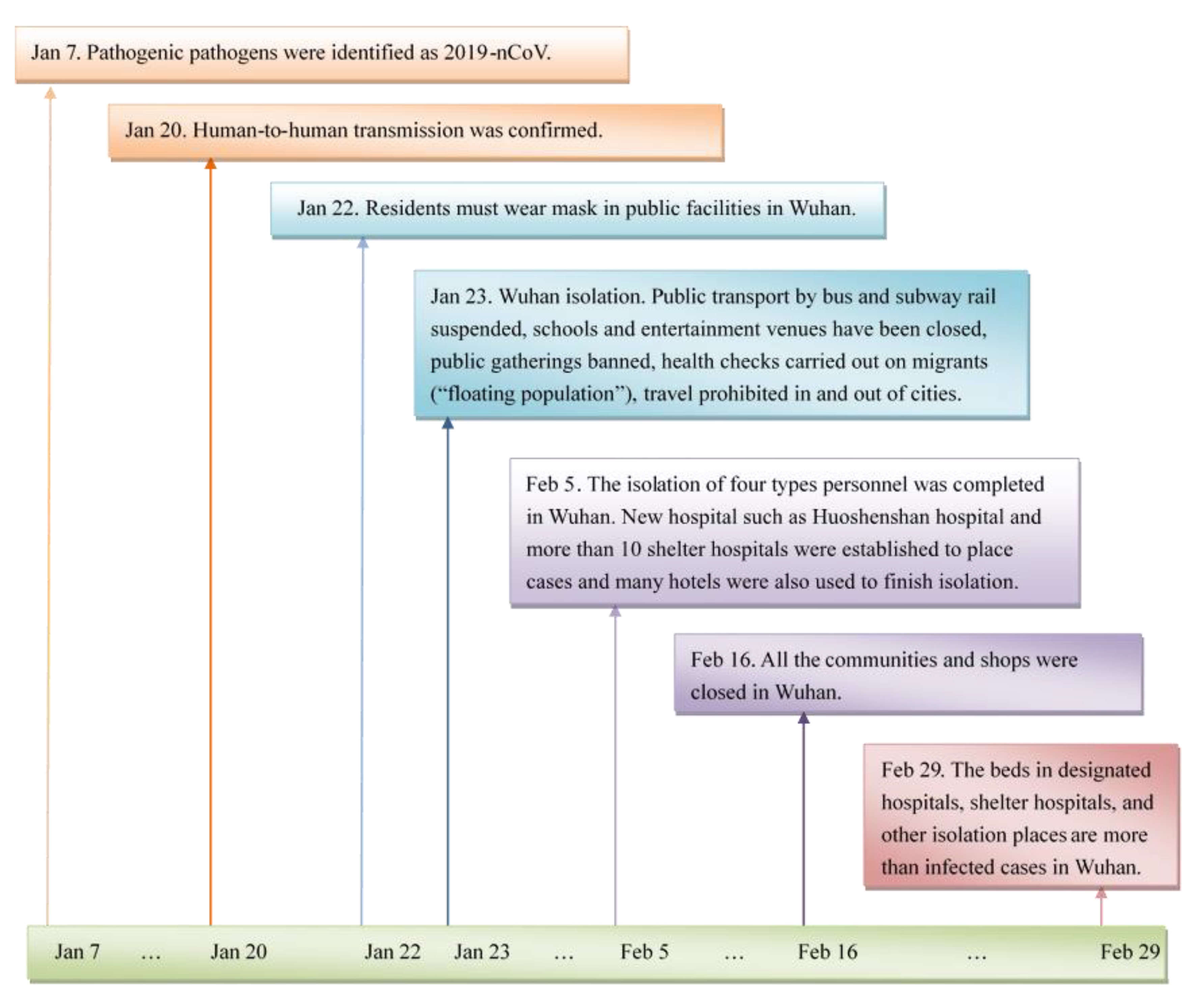


**e-Figure 2** The number of daily total confirmed cases in Wuhan until Mar 18, 2020


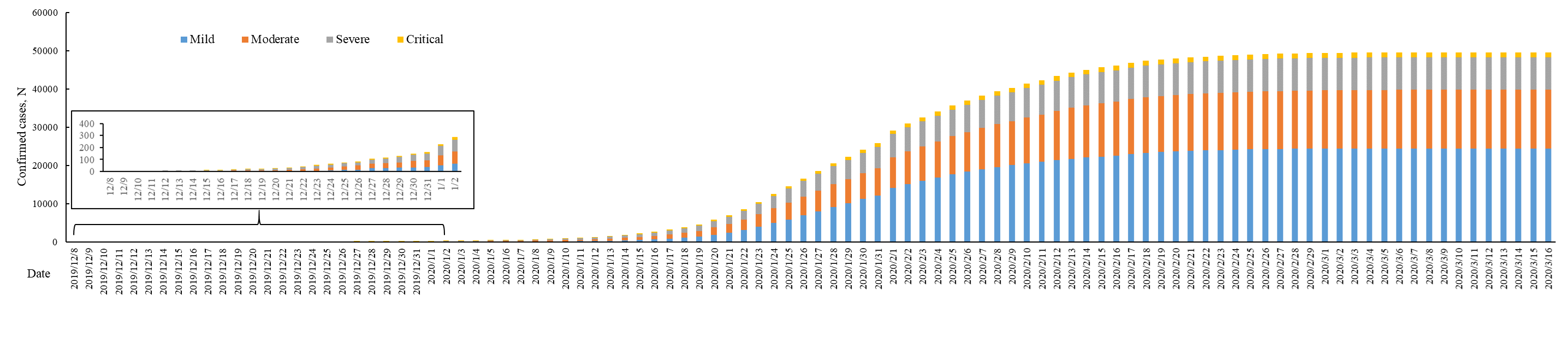


**e-Figure 3** The number of total confirmed cases in Wuhan: A, before Jan 23, 2020; B, until Feb 4, 2020; C, until Feb 15, 2020; D, until Mar 18, 2020.


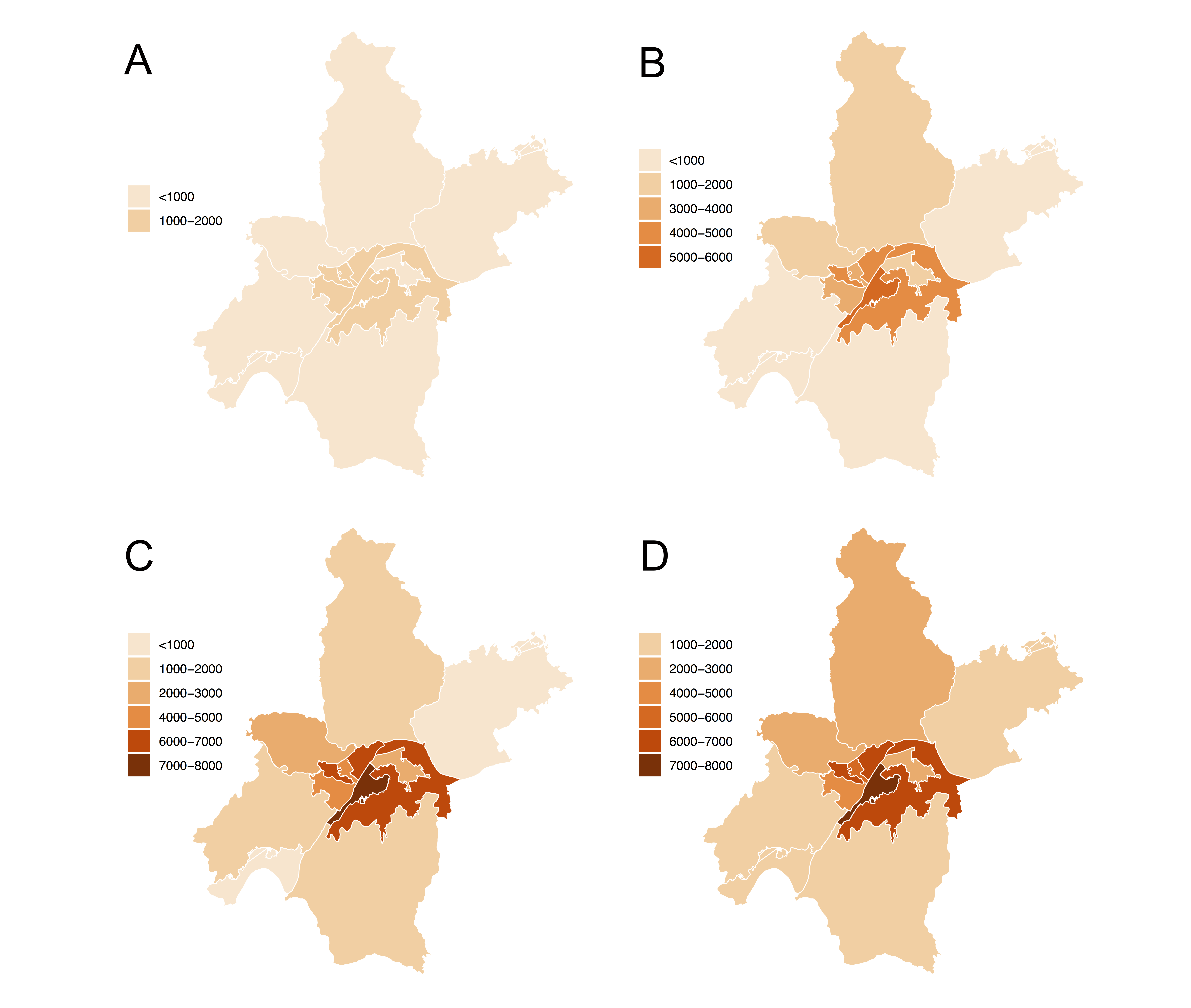


**e-Figure 4** The number of daily new confirmed cases by sex in Wuhan until Mar 18, 2020: (A) male; (B) female

(A)


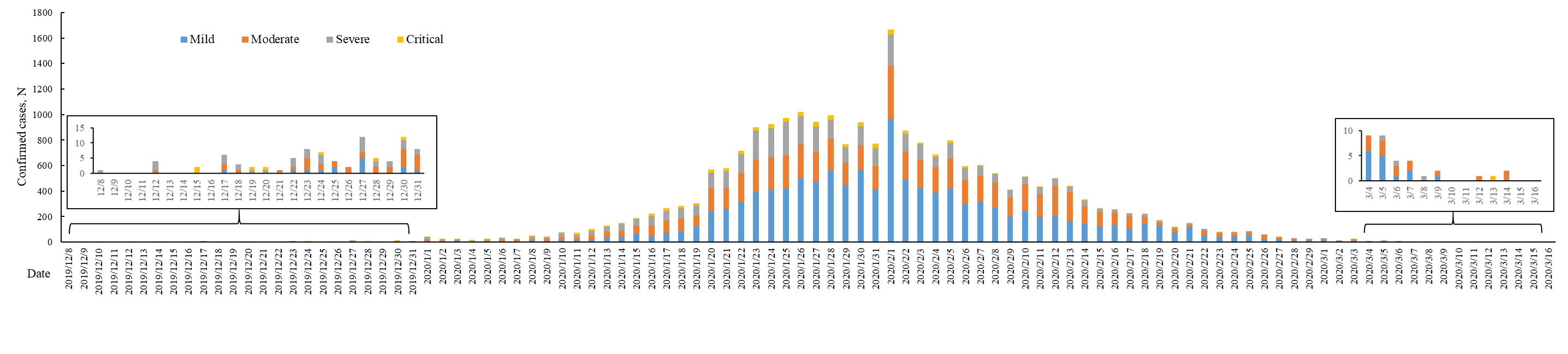


(B)


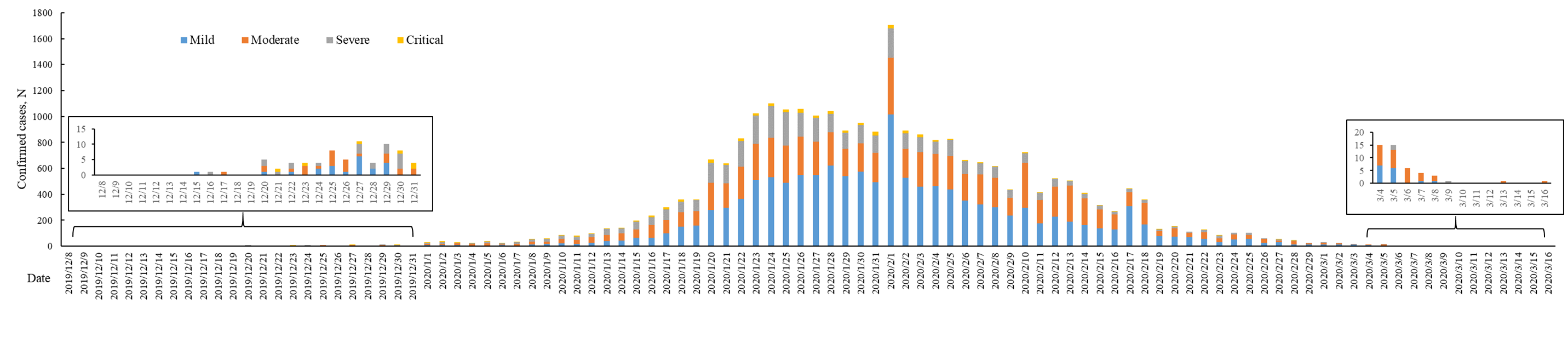


**e-Figure 5** The number of daily new confirmed cases by age group in Wuhan until Mar 18, 2020: (A) 0-≤18; (B) 19-≤40; (C) 41-60; (D) 61-80; (E) ≥81

(A)


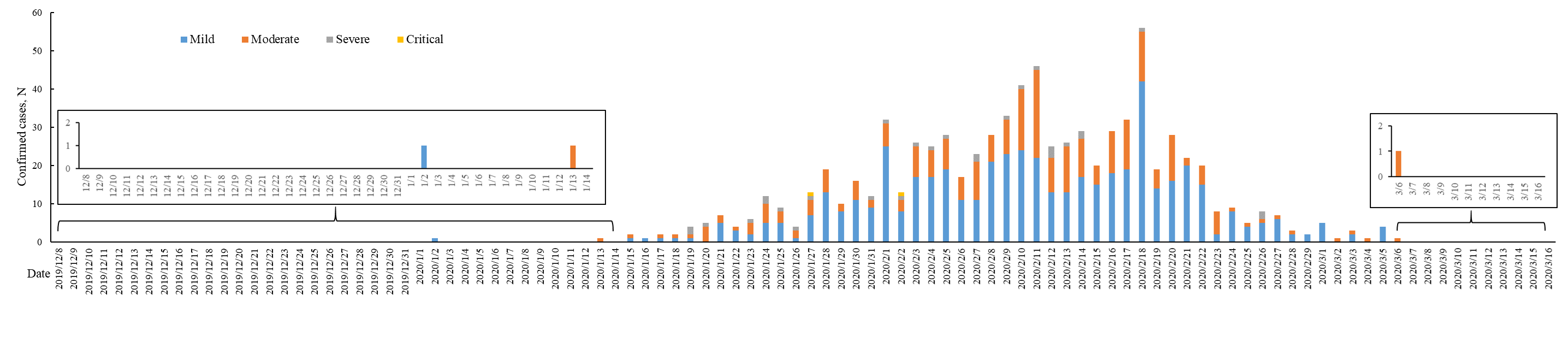


(B)


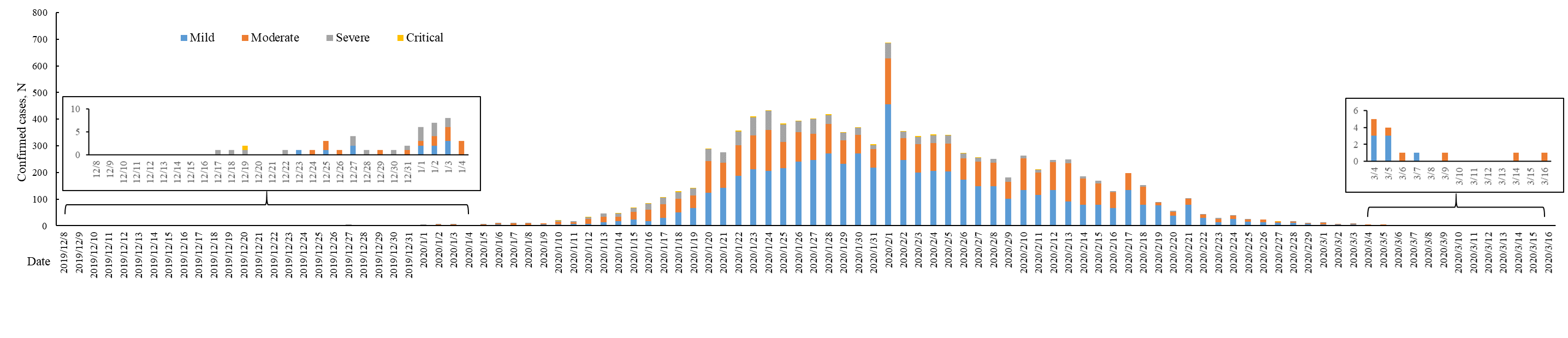


(C)


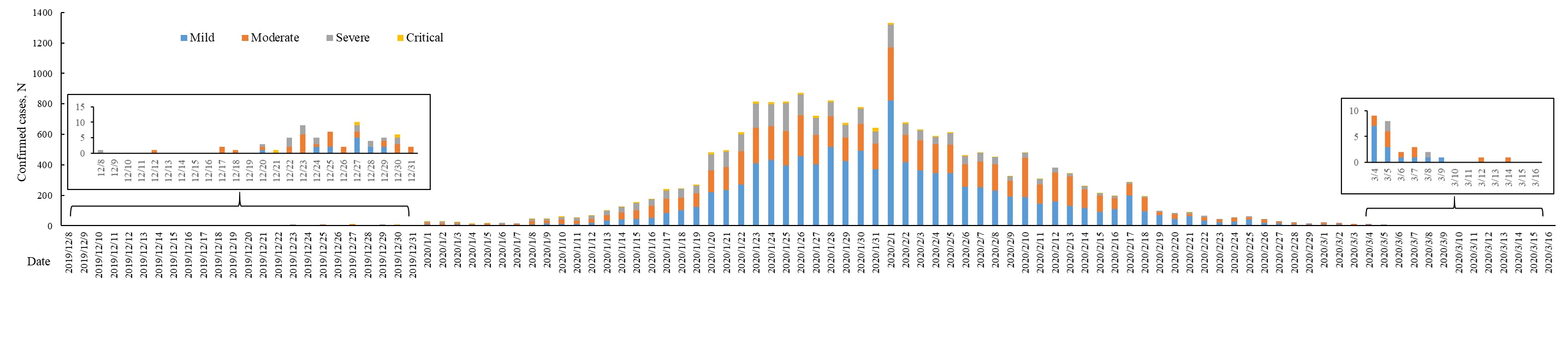


(D)


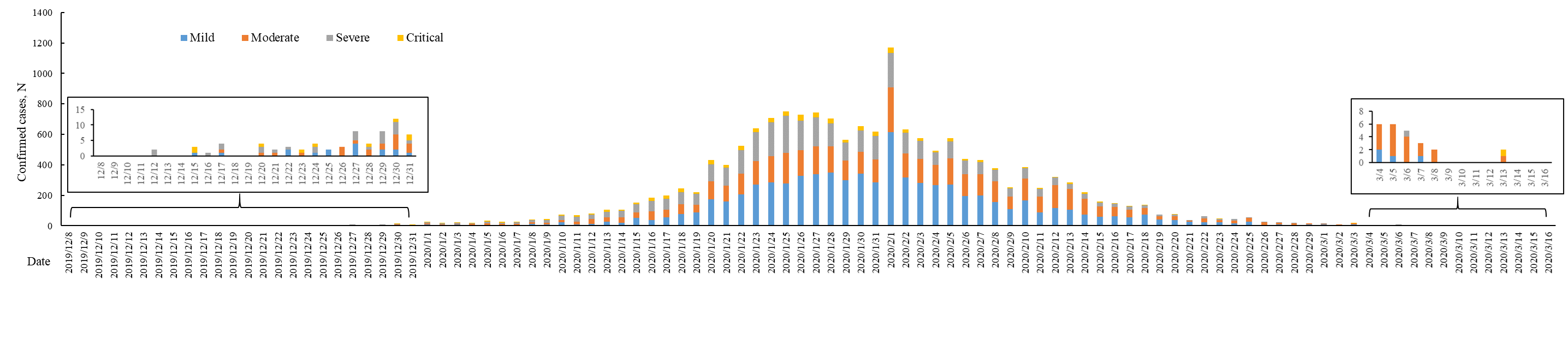


(E)


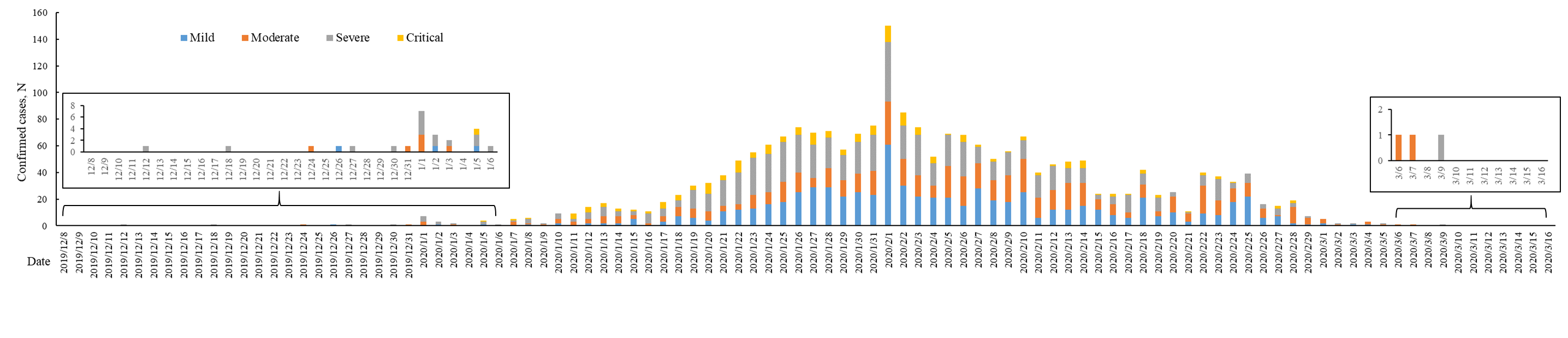


**e-Figure 6** The number of daily total confirmed cases by sex in Wuhan until Mar 18, 2020: (A) male; (B) female

(A)


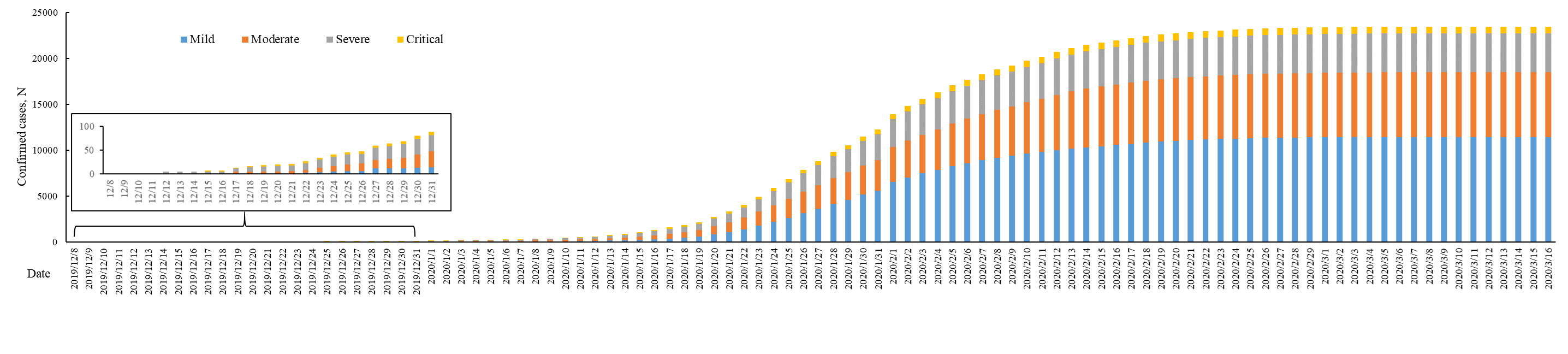


(B)


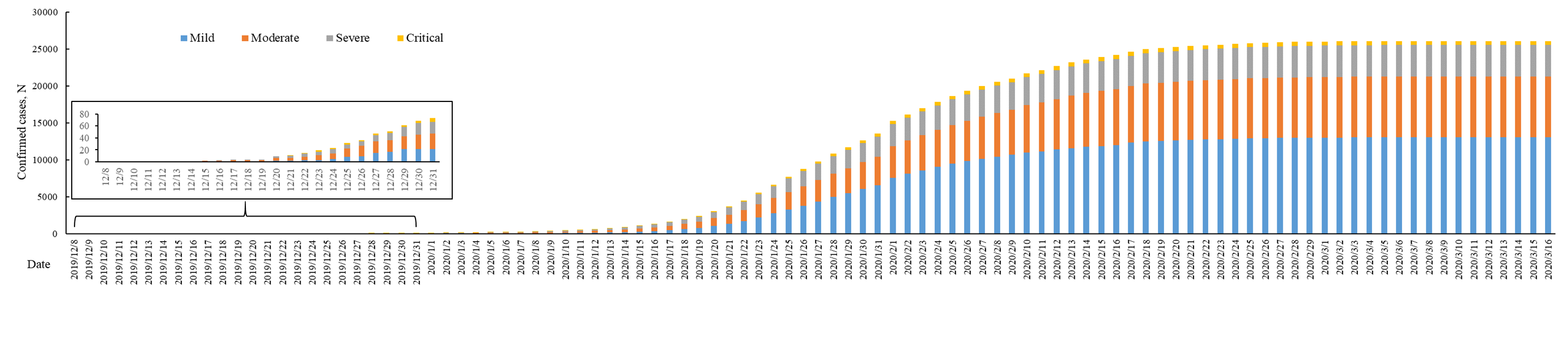


**e-Figure 7** The number of daily total confirmed cases by age group in Wuhan until Mar 18, 2020: (A) 0-≤18; (B) 19-≤40; (C) 41-60; (D) 61-80; (E) ≥81

(A)


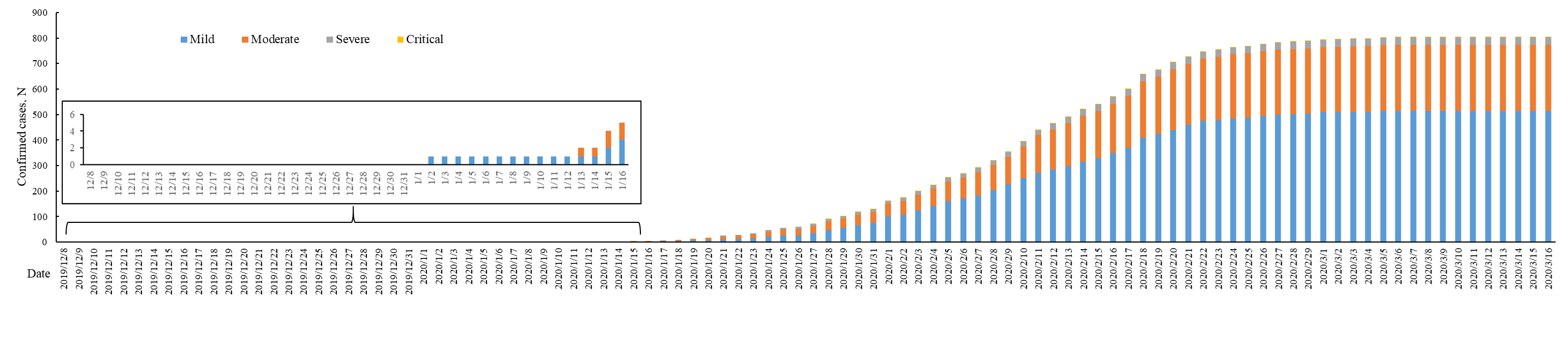


(B)


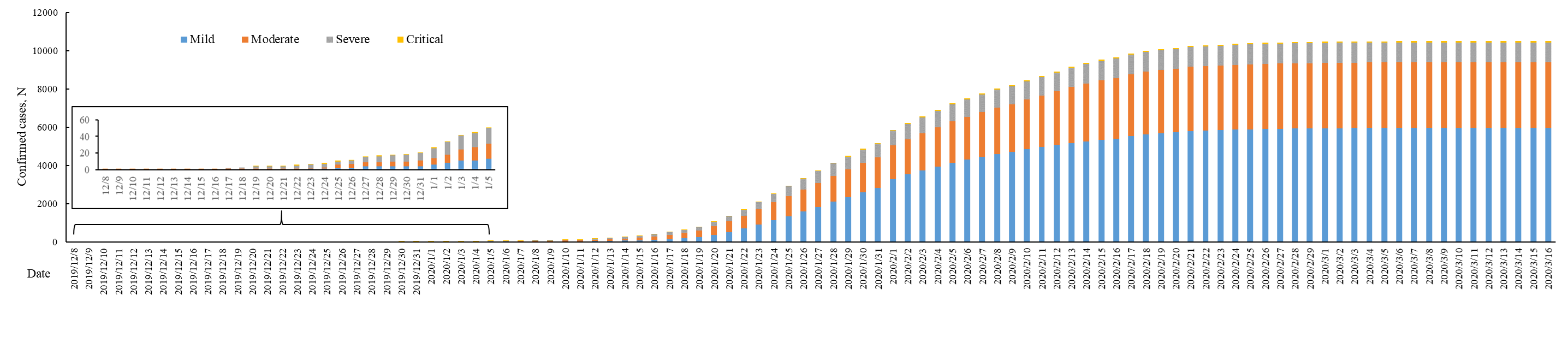


(C)


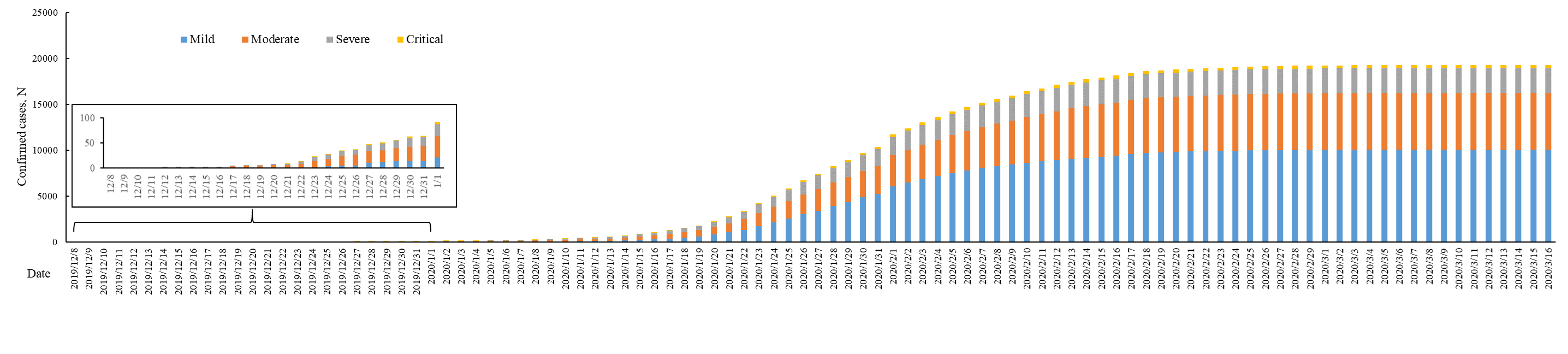


(D)


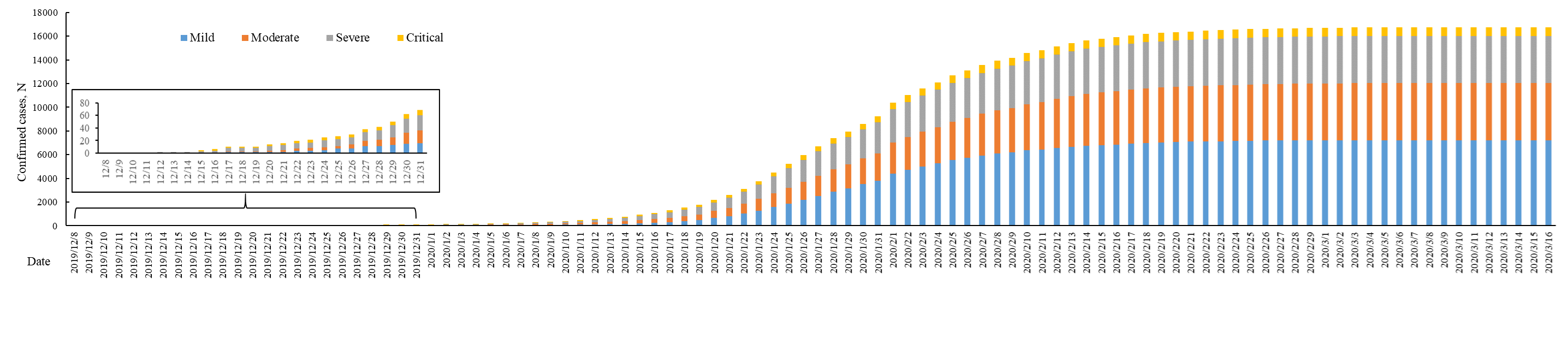


(E)


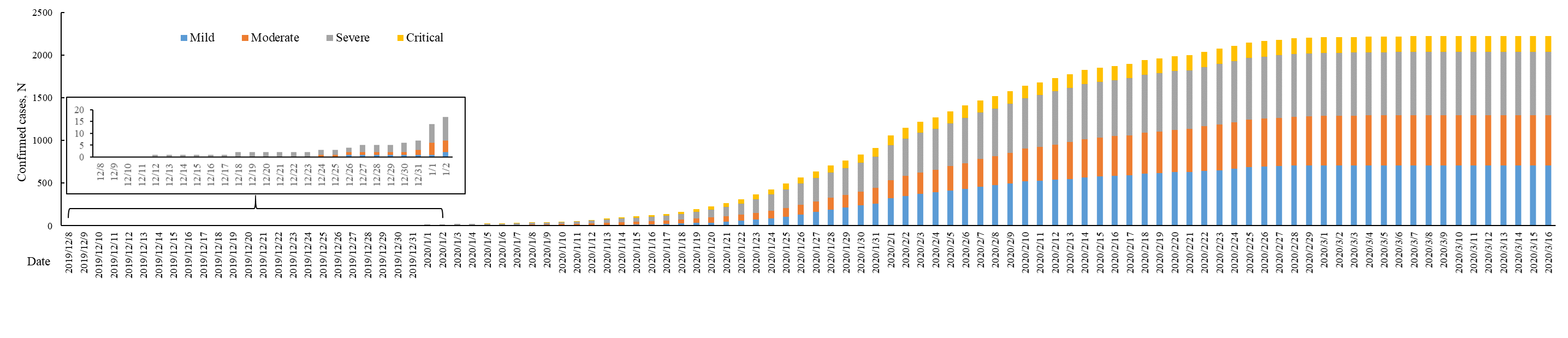

Supplement: Supplementary file 1 — Additional file 1: e-Table 1. Characteristics of different severity types of cases in Wuhan until Mar 18, 2020. e-Table 2. Association of different severity types of cases with the population in Wuhan. e-Figure 1. Dates of discovery of COVID-19, and of the key implementation of control measures in Wuhan. e-Figure 2. The number of daily total confirmed cases in Wuhan until Mar 18, 2020. e-Figure 3. The number of total confirmed cases in Wuhan: A, before Jan 23, 2020; B, until Feb 4, 2020; C, until Feb 15, 2020; D, until Mar 18, 2020. e-Figure 4. The number of daily new confirmed cases by sex in Wuhan until Mar 18, 2020: (A) male; (B) female. e-Figure 5. The number of daily new confirmed cases by age group in Wuhan until Mar 18, 2020: (A) 0- ≤ 18; (B) 19- ≤ 40; (C) 41–60; (D) 61–80; (E) ≥81. e-Figure 6. The number of daily total confirmed cases by sex in Wuhan until Mar 18, 2020: (A) male; (B) female. e-Figure 7. The number of daily total confirmed cases by age group in Wuhan until Mar 18, 2020: (A) 0- ≤ 18; (B) 19- ≤ 40; (C) 41–60; (D) 61–80; (E) ≥81. [file 12931_2020_1525_MOESM1_ESM.docx]
